# Ornamental plants associated with Buddhist figures in China

**DOI:** 10.1186/s13002-023-00595-3

**Published:** 2023-05-25

**Authors:** Xiaodan Xu, Chengmin Yan, Zhiying Ma, Qi Wang, Jie Zhao, Rui Zhang, Luyao He, Wei Zheng

**Affiliations:** Laboratory of Landscape Plants and Ecology, Faculty of Architecture and City Planning, Kunming University of Science and Technology, Kunming, 650504 Yunnan China

**Keywords:** Sakyamuni Buddha, Asoka tree, Bodhi tree, Sal tree, Substitute species

## Abstract

**Background:**

In China, many ornamental plants associated with Buddhist figures, including the Sakyamuni, Bodhisattva, and Arhat, were grown and worshiped because of their cultural and religious significance. However, the systematic collation and ethnobotanical information about these culturally important plants have yet to be fully understood.

**Methods:**

Online information was collected from 93 e-commercial platforms for ornamental plants all over China. Field sampling was conducted in 16 ornamental markets and 163 Buddhist temples using key informant interviews and participatory observation with traders, tourists, and local disciples. The types, distributions, and associated characteristics of the screened plants were summarized and the evolving characteristics of these ornamental plants were analyzed.

**Results:**

A total of 60 ornamental plants, including six varieties and one subspecies, were screened, of which 43 species were associated with Sakyamuni, 13 with Bodhisattva, and four with Arhat. Among the 60 species, three were regarded as the Asoka tree related to Buddha's birth, ten as the Bodhi tree connected to Buddha's enlightenment, three as the Sal tree associated with Buddha's nirvana, nine were related to Buddha’s head, belly, or hand, and 18 were connected with Buddha as lotus throne, bamboo monastery, or Bodhi beads. The evolving characteristics of these ornamental plants primarily constituted the substitution of the original plants by similar native plant species, followed by the introduced species with comparable morphology to the Buddhist figures.

**Conclusions:**

People grow ornamental plants associated with Buddhist figures to reflect their love and praise for plants and Buddha. The association between the ornamental plants and Buddhist figures will aid the inheritance of Buddhist culture and promote ornamental plants in the commercial market. Thus, the ethnobotany of ornamental plants associated with Buddhist figures can serve as a basis for future investigation of modern Buddhist culture.

## Background

Various plant species have been worshiped and praised worldwide for their cultural significance [1]. Sakyamuni was born in Kapilavatsu, Nepal, in about 565 B.C., and later became Buddha, the founder of the Buddhist religion [2]. Sakyamuni's life is believed to be closely associated with three different types of trees. He was born under the Asoka tree (*Saraca asoca* (Roxb.) W.J.de Wilde), enlightened under the Bodhi tree (*Ficus religiosa* L.), and gained nirvana under the Sal tree (*Shorea robusta* Gaertn.) [2, 3]. Furthermore, Buddha loved the flower of *Nymphaea tetragona* and often sat on a lotus throne while lecturing [2]. Buddha’s bamboo monastery in the Patna region of Bihar, India, was the first building dedicated to Buddhists [2, 3].

Along with the spread of Buddhism from its origin, the elements of Buddhism, such as worshiping trees, Asoka, Bodhi, and Sal trees, have been planted around Buddha temples to commemorate Buddha [2, 3]. These trees constituted an important component of the temple landscapes. Later, other ornamental plants, including bamboo and lotus-like flowers, were also grown by Buddhist disciples to commemorate well-known Buddhist figures, such as Sakyamuni Buddha, Buddhist Bodhisattva, and Arhat (the self-conscious figure who is lower than Buddha and Bodhisattva in Mahayana Buddhism) [4, 5]. In China, more and more ornamental plants were gradually planted and linked to Buddhist figures. These plants were commemorated and prayed for blessings in daily life and have become the carrier of Buddhist culture [3].

Buddhism spread northward from its origin, northern India, to China during the Han Dynasty (about 67 A.D.). Then, it moved to Mongolia, North Korea, South Korea, and Japan [4]. Buddhism practiced in East Asia (north of India) is often known as Northern or Mahayana Buddhism [4]. As Buddhism expanded north, different plant species gradually replaced the associated ornamental plants from the south due to different climatic conditions and aesthetic value [6]. Despite the widespread adoption of various ornamental plants to commemorate Buddhist figures, the systematic collation and diversity of these plants have yet to be fully understood. This paper summarized the ornamental plants associated with Buddhist figures in China to understand their evolving characteristics.

## Methods

**Fig. 1:**
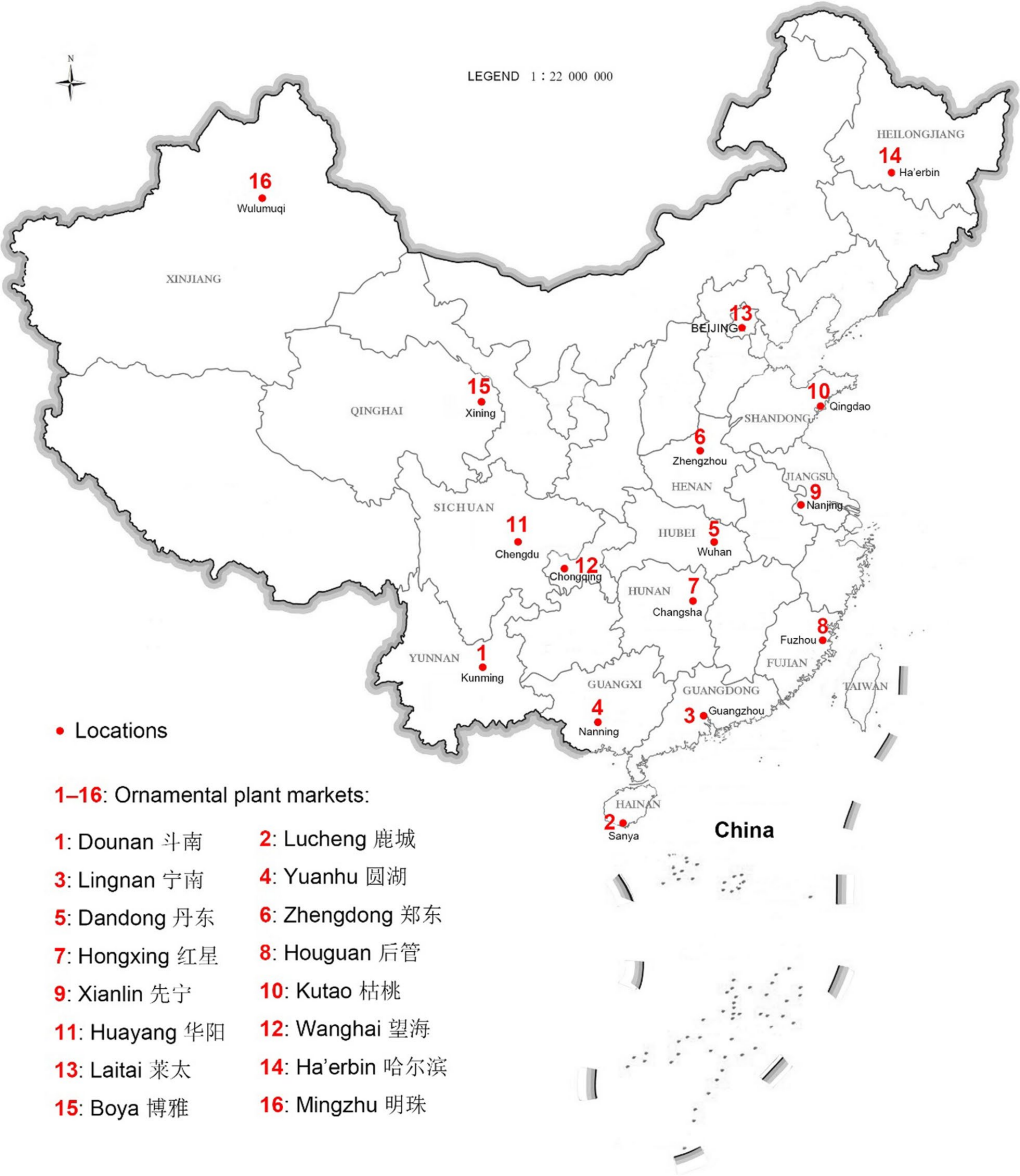
Locations of the investigated ornamental plant markets in China

Online information was collected from 93 e-commercial platforms for ornamental plants used in horticulture and landscaping all over China. Field sampling was conducted in 16 ornamental markets and 163 Buddhist temples from September 2021 to March 2023, using key informant interviews and participatory observation with traders, tourists, and disciples in local temples. Dounan flower market (29°39′N, 91°7′E, Fig. 1) in Kunming, Yunnan, received particular attention during the surveys because the market is one of the biggest flower markets (fresh cut flowers and potted flowers) in Buddhist regions. Other markets (Fig. 1), namely the Lucheng flower market in Sanya (Hainan), Lingnan in Guangzhou (Guangdong), and Yuanhu in Nanning (Guangxi) from South (S) China, were considered to collect data. In addition, data were collected from the Dandong flower market in Wuhan (Hubei), Zhengdong in Zhengzhou (Henan), and Hongxing in Changsha (Hunan) from Central (C) China; Houguan flower market in Fuzhou (Fujian), Xianlin in Nanjing (Jiangsu), and Kutao in Qingdao (Shandong) from East (E) China; and Huayang flower market in Chengdu (Sichuan) and Wanghai in Chongqing from Southwest (SW) China. Laitai flower market in Beijing from North (N) China; Ha’erbin flower market in Ha’erbin (Heilongjiang) from Northeast (NE) China and Boya flower market in Xining (Qinghai) and Mingzhu in Wulumuqi (Xinjiang) from Northwest (NW) China, were also considered for data collection.

All the ornamental plants were photographed and identified to species using standard literature according to morphological characters and geographical origins [7]. The types (trees, shrubs, herbs, and vines), associated characteristics (which figures, and how to associated), and multiple uses (ornamental, medicinal, edible, and economical) of the screened plants were summarized. The evolving characteristics of ornamental plants associated with Buddhist figures were also analyzed.

A detailed inventory of the substitutes for the Bodhi tree (*Ficus religiosa*, Fig. 1A) in temples was carried out to calculate an occurrence frequency (OF). For example, if *N* temples sold Bodhi tree type “A” (*N* ≥ 1), then the occurrence frequency of “A” is OF(*A*) = *N*/126 × 100%.

## Results and discussion

A total of 60 ornamental plant species (including six varieties and one subspecies) belonging to 37 families and 48 genera associated with Buddhist figures were identified from China (Table 1). Among the 60 plants, 25 were tree species (41.67%, 10 evergreen and 15 deciduous), 13 shrubs (21.67%, nine evergreen and four deciduous), 13 herbs (21.67%), and nine vines (15.00%). Thirty nine species (65.00%) were native to China, whereas 21 (35.00%) were introduced from other countries. Among the identified species, 43 species (71.67%) were associated with Shakyamuni Buddha, 13 (21.67%) with Bodhisattva, and four (6.67%) with Arhat. It can be concluded that Shakyamuni Buddha, who can guide human moral behavior and deter various evils [2], is the most popular Buddhist figure in China.

**Table 1:** Characteristics of ornamental plants associated with Buddhist figures in China

No.	Scientific name	Family name	Vernacular name	Buddhist figures and associated characteristics	Use(s)	Type	Origin
1	*Saraca asoca* (Roxb.) W.J.de Wilde	Fabaceae	Wu you shu 无忧树	Buddha was born under the Asoka tree	Ornamental, medicinal	Evergreen tree	Tropical Asia
2	*Saraca dives* Pierre	Fabaceae	Zhong guo wu you shu 中国无忧树	Diversified species as the Asoka tree	Ornamental, medicinal	Evergreen tree	Southeast Asia
3	*Sapindus saponaria* L	Sapindaceae	Wu huan zi 无患子	Substitute for the Asoka tree	Ornamental, medicinal	Deciduous tree	SE Asia and Japan
4	*Ficus religiosa* L	Moraceae	Pu ti shu 菩提树	Buddha Enlighted under the Bodhi tree	Ornamental	Evergreen tree	Himalayas, S and E Asia
5	*Syringa reticulata* (Blume) H. Hara	Oleaceae	Bao ma ding xiang 暴马丁香	Substitute for the Bodhi tree	Ornamental, perfume	Deciduous shrub or small tree	N, NE and NW China
6	*Syringa oblata* Lindl	Oleaceae	Ding xiang 丁香	Substitute for the Bodhi tree	Ornamental	Deciduous shrub or small tree	N, NE and NW China
7	*Ginkgo biloba* L	Ginkgoaceae	Yin xing 银杏	Substitute for the Bodhi tree	Ornamental, medicinal, edible, timber	Deciduous tree	E China
8	*Celtis bungeana* Bl	Ulmaceae	Pu shu 朴树	Substitute for the Bodhi tree	Ornamental	Deciduous tree	China and N Korea
9	*Tilia mongolica* Maxim	Tiliaceae	Meng duan 蒙椴	Substitute for the Bodhi tree	Ornamental, nectar source	Deciduous tree	N and NE China
10	*Tilia miqueliana* Maxim	Tiliaceae	Nan jing duan 南京椴	Substitute for the Bodhi tree	Ornamental, nectar source	Deciduous tree	E China and Japan
11	*Tilia henryana* var. *subglabra* V.Engl	Tiliaceae	Nuo mi duan 糯米椴	Substitute for the Bodhi tree	Ornamental, nectar source	Deciduous tree	C, N and NE China
12	*Catalpa bungei* C. A. Mey	Bignoniaceae	Qiu 楸	Substitute for the Bodhi tree	Ornamental, medicinal, edible, timber	Deciduous tree	C and N China
13	*Ficus virens* Aiton	Moraceae	Huang ge shu 黄葛树	Substitute for the Bodhi tree	Ornamental, medicinal, edible	Deciduous tree	S and SE Asia
14	*Shorea robusta* C. F. Gaertner	Dipterocarpaceae	Suo luo shu 娑罗树	Buddha was nirvana under the Sal tree	Ornamental, perfume, medicinal, timber	Deciduous tree	India and Malay Peninsula
15	*Aesculus chinensis* Bunge	Hippocastanaceae	Qi ye shu 七叶树	Substitute for the Sal tree	Ornamental, medicinal	Deciduous tree	C and E China
16	*Couroupita guianensis* Aubl	Lecythidaceae	Pao dan shu 炮弹树	Substitute for the Sal tree	Ornamental, medicinal, perfume	Evergreen tree	S America and S Caribbean
17	*Annona squamosa* L	Annonaceae	Fo tou guo 佛头果	Fruit looks like Buddha's head	Fruit, ornamental, medicinal, papermaking	Deciduous small tree	Tropical Americas
18	*Larryleachia cactiformis* (Hook.) Plowes	Apocynaceae	Fo tou yu佛头玉	Succulent stem looks like the spiral hair of Buddha’s head	Ornamental	Perennial succulent herb	S Africa, Namibia
19	*Phyllostachys* *edulis* “Heterocycla”	Poaceae	Fo mian zhu 佛面竹	Trunk looks like Buddha's face	Ornamental, artwork materials	Arbor or shrubby bamboo	C and E China
20	*Jatropha podagrica* Hook	Euphorbiaceae	Fo du shu 佛肚树	Trunk looks like Buddha’s belly	Ornamental, medicinal	Deciduous shrub	C America
21	*Brachychiton rupestris* (Lindl.) K. Schum	Malvaceae	Ao zhou fo du shu 澳洲佛肚树	Trunk looks like Buddha’s belly	Ornamental	Deciduous or semi-deciduous tree	Australia
22	*Bambusa ventricosa* McClure	Poaceae	Fo du zhu 佛肚竹	Trunk looks like Buddha’s belly	Ornamental	Arbor or shrubby bamboo	S China
23	*Citrus medica* “Fingered”	Rutaceae	Fo shou 佛手	Fruit looks like Buddha's hand	Ornamental, medicinal	Evergreen tree or shrub	S China
24	*Sechium edule* (Jacq.) Swartz	Cucurbitaceae	Fo shou gua 佛手瓜	Fruit looks like Buddha's hand	Vegetable, ornamental, medicinal	Perennial grass vine	Mexico
25	*Sedum lineare* Thunb	Crassulaceae	Fo jia cao 佛甲草	Leaf looks like Buddha's fingernail	Ornamental, medicinal	Perennial succulent herb	C, S, SW and SE China
26	*Nymphaea tetragona* Georgi	Nymphaeaceae	Shui lian 睡莲	Associated with Buddha’s lotus throne	Ornamental	Perennial aquatic herb	Tropical SE Asia
27	*Nelumbo nucifera* Gaertn	Nelumbonaceae	Lian/He hua 莲/荷花	Diversified species with lotus throne	Ornamental, edible, medicinal	Perennial aquatic herb	Tropical Asia
28	*Curcuma alismatifolia* Gagnep	Zingiberaceae	Jiang he hua 姜荷花	Diversified species with lotus throne	Ornamental	Perennial bulbous herb	Chiang Mai, Thailand
29	*Musella lasiocarpa* (Franchet) C. Y. Wu ex H. W. Li	Musaceae	Di yong jin lian 地涌金莲	Diversified species with lotus throne	Ornamental, edible, medicinal	Perennial herb	Yunnan, China
30	*Lirianthe delavayi* (Franchet) N. H. Xia & C. Y. Wu	Magnoliaceae	You tan hua 优昙花	Diversified species with lotus throne	Ornamental, medicinal	Evergreen tree	SW China
31	*Manglietia* *insignis *(Wall.) Blume	Magnoliaceae	Hong hua mu lian 红花木莲	Diversified species with lotus throne	Ornamental, medicinal, timber	Evergreen tree	SW China, N Myanmar, and Thailand
32	*Camellia reticulata* Lindl	Theaceae	Dian shan cha 滇山茶	Diversified species with lotus throne	Ornamental, oil	Evergreen tree	SW China
33	*Protea cynaroides* L	Proteaceae	Pu ti hua 菩提花	Diversified species with lotus throne	Ornamental, medicinal	Evergreen shrub	S Africa
34	*Clematis florida* Thunb	Ranunculaceae	Tie xian lian 铁线莲	Diversified species with lotus throne	Ornamental, medicinal	Herbaceous vine	China and Japan
35	*Tropaeolum majus* L	Tropaeolaceae	Han jin lian 旱金莲	Diversified species with lotus throne	Ornamental, medicinal, edible	Semi-creeping herb	S America
36	*Trollius chinensis* Bunge	Ranunculaceae	Jin lian hua 金莲花	Diversified species with lotus throne	Ornamental, medicinal	Annual or perennial herb	N and NE China
37	*Passiflora caerulea* L	Passifloraceae	Xi fan lian 西番莲	Diversified species with lotus throne	Ornamental	Perennial herbaceous vine	Tropical America
38	*Passiflora coccinea* Aubl	Passifloraceae	Hong hua xi fan lian 红花西番莲	Diversified species with lotus throne	Ornamental, medicinal, edible	Perennial evergreen climbing woody vine	Les islands
39	*Echeveria secunda* Booth ex Lindl	Crassulaceae	Shi lian hua 石莲花	Diversified species with lotus throne	Ornamental	Perennial evergreen succulent herb	Tropical America
40	*Senecio rowleyanus* H. Jacobsen	Asteraceae	Fo zhu 佛珠	Leaves look like Bodhi beads	Ornamental	Perennial evergreen creeping succulent herb	S Africa
41	*Phyllostachys sulphurea* (Carr.) A. et C. Riv	Poaceae	Jin zhu 金竹	Associated with Buddha’s bamboo monastery	Ornamental, papermaking, edible	Arbor or shrubby bamboo	C China
42	*Chimonobambusa quadrangularis* (Fenzi) Makino	Poaceae	Fang zhu 方竹	Associated with Buddha’s bamboo monastery	Ornamental, walking sticks, edible	Shrubby bamboo	S and SW China
43	*Chimonobambusa tumidissinoda* Hsueh & T. P. Yi ex Ohrnberger	Poaceae	Qiong zhu 筇竹	Associated with Buddha’s bamboo monastery	Ornamental, walking sticks, edible	Shrubby bamboo	SW China
44	*Sempervivum tectorum* L	Crassulaceae	Guan yin lian 观音莲	Associated with the lotus throne of Bodhisattva Guanyin	Ornamental	Perennial evergreen succulent herb	Caucasus in Europe and Asia
45	*Phyllostachys nigra* (Lodd.) Munro	Poaceae	Zi zhu 紫竹	Associated with Guanyin’s bamboo monastery	Ornamental, artwork materials	Arbor or shrubby bamboo	C and S China
46	*Bambusa multiplex* var. *riviereorum* R.Maire	Poaceae	Guan yin zhu 观音竹	Diversified species with Guanyin’s bamboo monastery	Ornamental	shrubby bamboo	S China
47	*Rhapis excelsa* (Thunb.) Henry ex Rehd	Arecaceae	Zong zhu 棕竹	Diversified species with Guanyin’s bamboo monastery	Ornamental	Evergreen shrub	SE Asia
48	*Dracaena sanderiana* Sander	Asparagaceae	Fu gui zhu 富贵竹	Diversified species with Guanyin’s bamboo monastery	Ornamental	Evergreen subshrub	Tropical Africa and Asia
49	*Salix babylonica* L	Salicaceae	Chui liu 垂柳	Associated with the holy branches in Guanyin’s bottle	Ornamental, medicinal, timber	Deciduous tree	C China
50	*Salix matsudana* Koidz	Salicaceae	Han liu 旱柳	Associated with the holy branches in Guanyin’s bottle	Ornamental, medicinal, timber, forage	Deciduous tree	China, Korea, and Japan
51	*Tamarix chinensis* Lour	Tamaricaceae	Cheng liu 柽柳	Associated with the holy branches in Guanyin’s bottle	Ornamental, medicinal	Deciduous tree	Mongolia, NW and N China
52	*Reineckea carnea* (Andrews) Kunth	Liliaceae	Ji xiang cao 吉祥草	Associated with the holy branches in Guanyin’s bottle	Ornamental, medicinal	Perennial evergreen herb	C, E, SW China, and Japan
53	*Alocasia macrorrhizos* (Linnaeus) G. Don	Araceae	Di shui guan yin 滴水观音	Associated with the holy branches in Guanyin’s bottle	Ornamental, medicinal	Perennial evergreen herb	S America
54	*Ceiba chodatii* (Hassl.) Ravenna	Malvaceae	Mi le shu 弥勒树	Trunk looks like Bodhisattva Maitreya	Ornamental	Deciduous tree	Tropical S America
55	*Crinum asiaticum* var. *sinicum* (Roxb.ex Herb.) Baker	Amaryllidaceae	Wen shu lan 文殊兰	Name is homonymous to Bodhisattva Manjushri	Ornamental, medicinal	Perennial herb	Indonesia, Sumatra
56	*Gloriosa superba* L	Colchicaceae	Jia lan 嘉兰	Name is homonymous to Bodhisattva Sangharama	Ornamental, medicinal	Perennial climbing herb	Tropical Asia and Africa
57	*Podocarpus macrophyllus* (Thunb.) Sweet	Podocarpaceae	Luo han song 罗汉松	Seed and its handle looks like Arhat	Ornamental, medicinal	Evergreen tree	China and Japan
58	*Podocarpus forrestii* Craib et W. W. Smith	Podocarpaceae	Da li luo han song 大理罗汉松	Seed and its handle looks like Arhat	Ornamental	Evergreen herb	Dali of China
59	*Siraitia grosvenorii* (Swingle) C. Jeffrey ex Lu et Z. Y. Zhang	Cucurbitaceae	Luo han guo 罗汉果	Root looks like Arhat	Ornamental, medicinal	Perennial climbing herb	S China
60	*Musa basjoo* Sieb. et Zucc	Musaceae	Ba jiao 芭蕉	Name is homonymous to Arhat Plantain	Ornamental, medicinal	Perennial herb	Ryukyu islands

1 The spelling of Sanskrit terms does not use the diacritics; 2 Ranked by associated figures Sakyamuni Buddha (from birth, enlightenment, nirvana, body parts, to lotus throne, and bamboo monastery; from the original associated species to adaptations), Buddhist Bodhisattva, and Arhat, followed by species names alphabetically.

### Ornamental plants associated with Buddha

#### Ornamental plants associated with Buddha’s birth, enlightenment, and nirvana

Buddha was believed to be born under a *Saraca asoca* tree, regarded as the original Asoka tree [3] with drooping tender leaves that look like Buddhist kasaya. Asoka and its expanded species *S. dives*, originated from tropical Asia and grew in S China, but cannot perform well in C and N China because of different climatic conditions. In these regions, the local species *Sapindus saponaria* has been planted as an alternative to the original Asoka tree. For instance, a 250-year-old *S. saponaria* tree was cultivated in the Guanyin Temple, Xuchang (Henan Province). The Chinese name for *Sapindus*, “Wu huan zi”, means have children, which is equivalent to birth, like the Asoka tree. Like the Asoka tree, *S. saponaria* has pinnately compound leaves with similar leaflets. Additionally, the seeds of *S. saponaria* are regarded as Bodhi beads “Gui jian chou” [8].

**Fig. 2:**
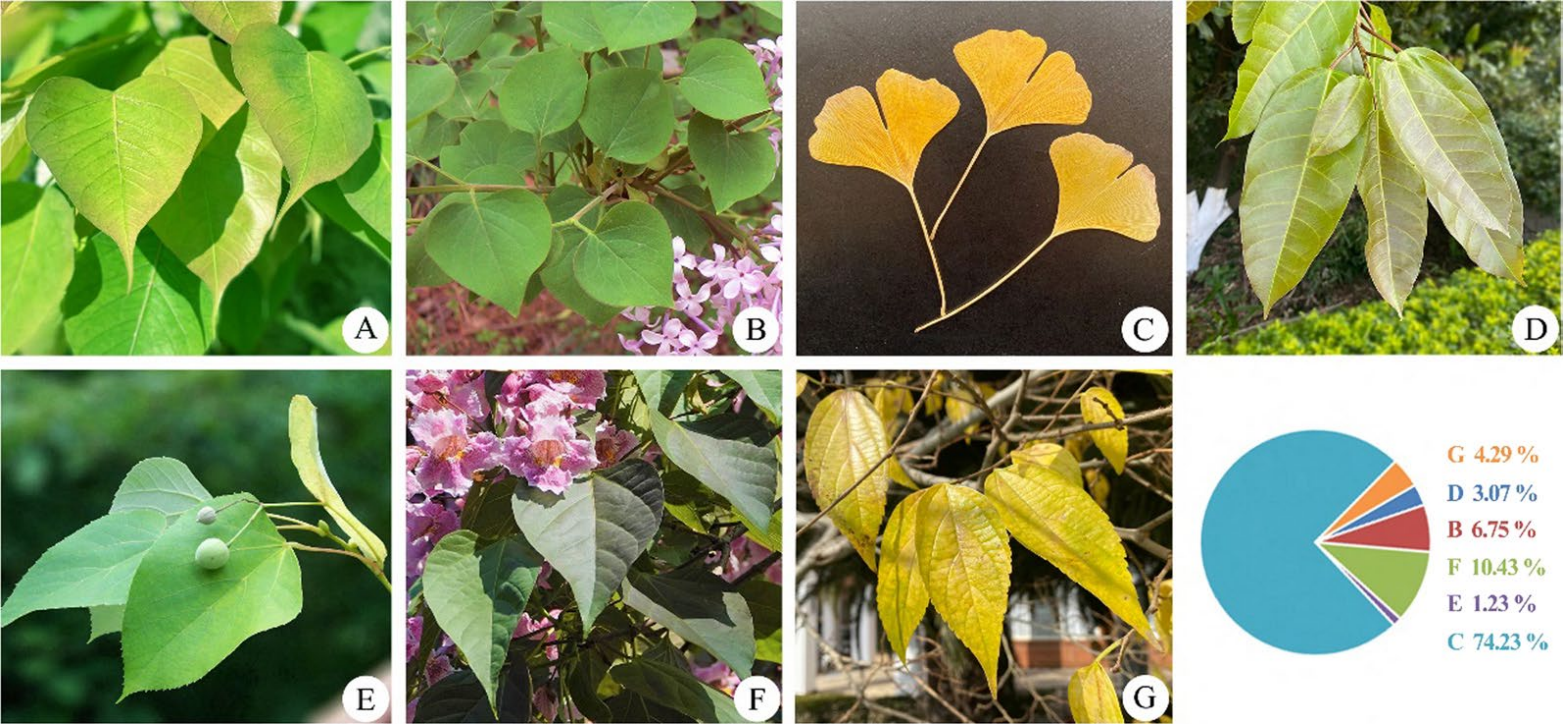
The original Bodhi tree and its substitutes. **A** *Ficus religiosa*; **B** *Syringa oblata*;** C** *Ginkgo biloba*; **D** *F. virens*; **E** *Tilia* sp.; **F** *Catalpa bungei*; and G: *Celtis bungeana*

The original Bodhi tree *Ficus religiosa*, under which the Buddha is believed to have meditated and attained enlightenment, has heart-shaped leaves with long drip tips (Fig. 2A), symbolizing magnanimity, enlightenment, and wisdom [9]. While the species can be planted in southern tropical and subtropical regions to commemorate Buddha and his enlightenment, it cannot grow well in temperate regions [5] and is substituted by climatically suitable local tree species with heart-shaped (or inverted heart-shaped) leaves (Table 1). For instance, *F. religiosa* was substituted by *Syringa* sp. (Fig. 2B) in N China, by *Celtis bungeana* (gold-yellow leaves in autumn, Fig. 2G) in N and E China, by *Tilia* sp. (Fig. 2E) and *Catalpa bungei* (Fig. 2F) in N, E, and C China, by *Ficus virens* (Fig. 2D) in SW China, and by *Ginkgo biloba* (golden leaves in autumn, Fig. 2C) in C, E, N, NW, and SW of China. These substitute species have been planted as ornamental trees in temples, urban green spaces, courtyards, and indoors as bonsai in China [10–13].

The most common alternative species used to replace the Bodhi tree was *Ginkgo biloba* (Fig. 2, OF = 74.23%), the Chinese Bodhi tree [14]. This substitution of the species dates back to at least 1400 years ago when a ginkgo tree was planted by Shimin Li, the second emperor of the Tang Dynasty, in Guanyin Temple, Xi'an, China. Similarly, in NW China, the substitute species *S. reticulata* for the Bodhi tree traced back to 1379 when the Taer Temple was built around a *S. reticulata* tree at Xining, Qinghai Province, China.

It is believed that Buddha attained nirvana under a *Shorea robusta* tree, which is native to the tropical rainforests of Asia [15] and cannot be grown in temperate areas. In the temperate regions, master Xuanzang brought *Aesculus chinensis* to Tongchuan City 1300 years ago to substitute *S. robusta* because Sakyamuni gathered his disciples for the first time under the *A. chinensis* tree [16]. Subsequently, the species was cultivated in Buddhist temples in N, E, and C China. For example, the Great Ci'en Temple in Xi’an, the Wofo Temple in Beijing, and the Lingyin Temple in Hangzhou, China, harbored more than 1000 years old *A. chinensis* trees commemorating Buddha. In S China, the *Couroupita guianensis* replaced the original Sal tree. Its flowers and fruits develop together all year round [17], implying reincarnation after death.

#### Ornamental plants associated with Buddha’s body

**Fig. 3:**
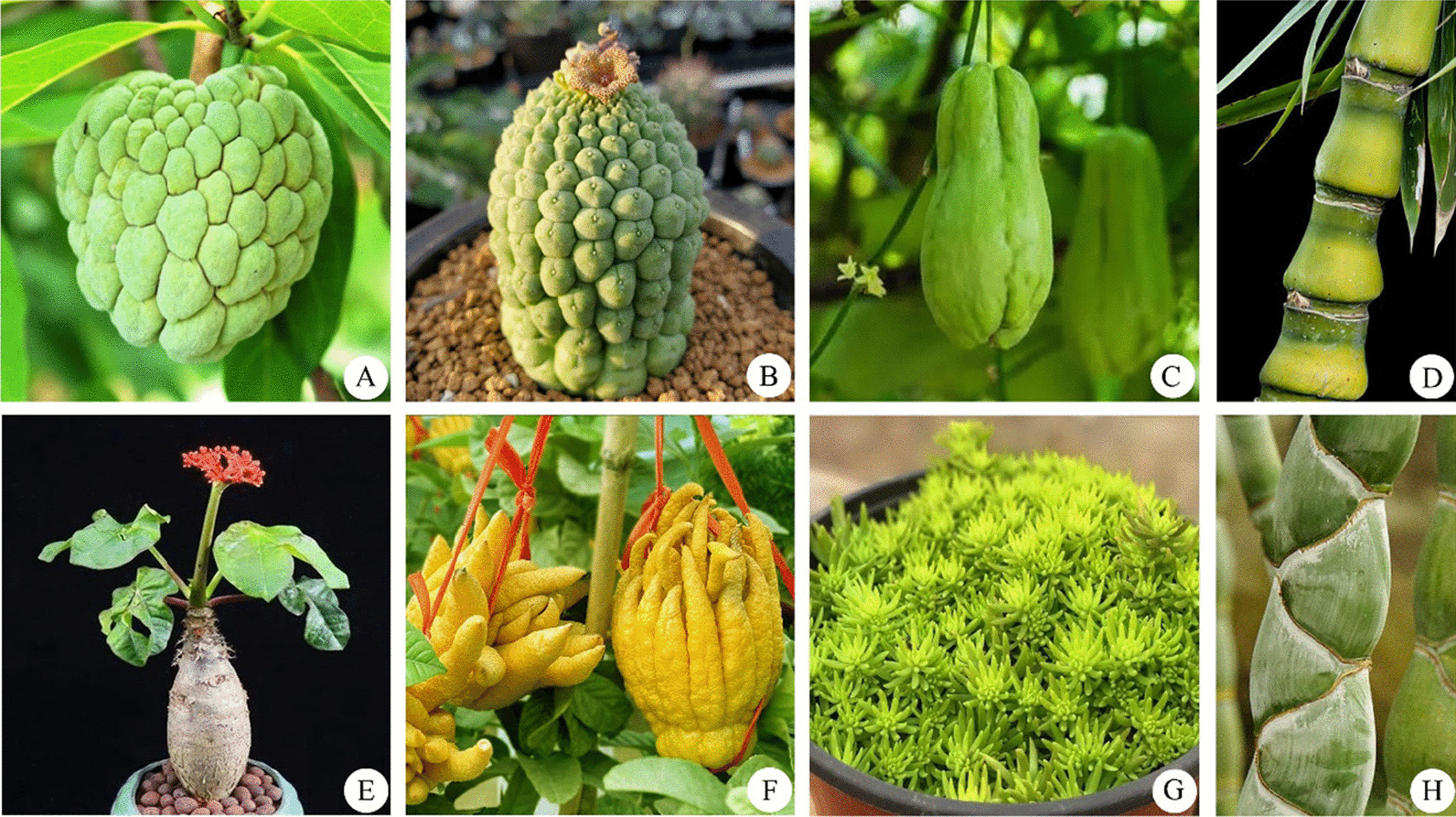
Ornamental plants associated with Buddha’s body. **A** *Annona squamosa*; **B** *Larryleachia cactiformis*; **C** *Sechium edule*; **D** *Bambusa ventricosa*; **E*** Jatropha podagrica*; **F** *Citrus medica* “Fingered”; **G** *Sedum lineare*; and **H** *Phyllostachys* *edulis* “Heterocycla”

During the spread of Buddhism, nine ornamental plants were adopted to commemorate Buddha's body. For instance, the adoption of *Annona squamosal* (Fig. 3A) was linked to its fruit resembling Buddha’s head. Similarly, the succulent stem of *Larryleachia cactiformis* (Fig. 3B) and the swollen stem of *Phyllostachys* *edulis* “Heterocycla” (Fig. 3H) resembled the spiral hair of Buddha’s head and face, respectively, and were adopted to commemorate Buddha’s body. Some other species adopted to commemorate Buddha included *Bambusa ventricosa* (Fig. 3D), *Jatropha podagrica* (Fig. 3E), and *Brachychiton rupestris*, as their swollen stems resemble Buddha’s belly. Moreover, the fruit of *Sechium edule* (Fig. 3C) and *Citrus medica* “Fingered” (Fig. 3F) fruits looked similar to Buddha’s closed and open hands, respectively, and were adopted during the expansion of Buddhism to commemorate Buddha. In addition, the leaves of *Sedum lineare* (Fig. 3G) resemble Buddha’s fingernails, and the plant was adopted to worship Buddha. Besides adopting to celebrate Buddha’s body, these plant species were widely used as potted ornamentals and planted across the urban landscape and agricultural parks.

#### Ornamental plants associated with Buddha as lotus throne, bamboo monastery, and Bodhi beads

**Fig. 4:**
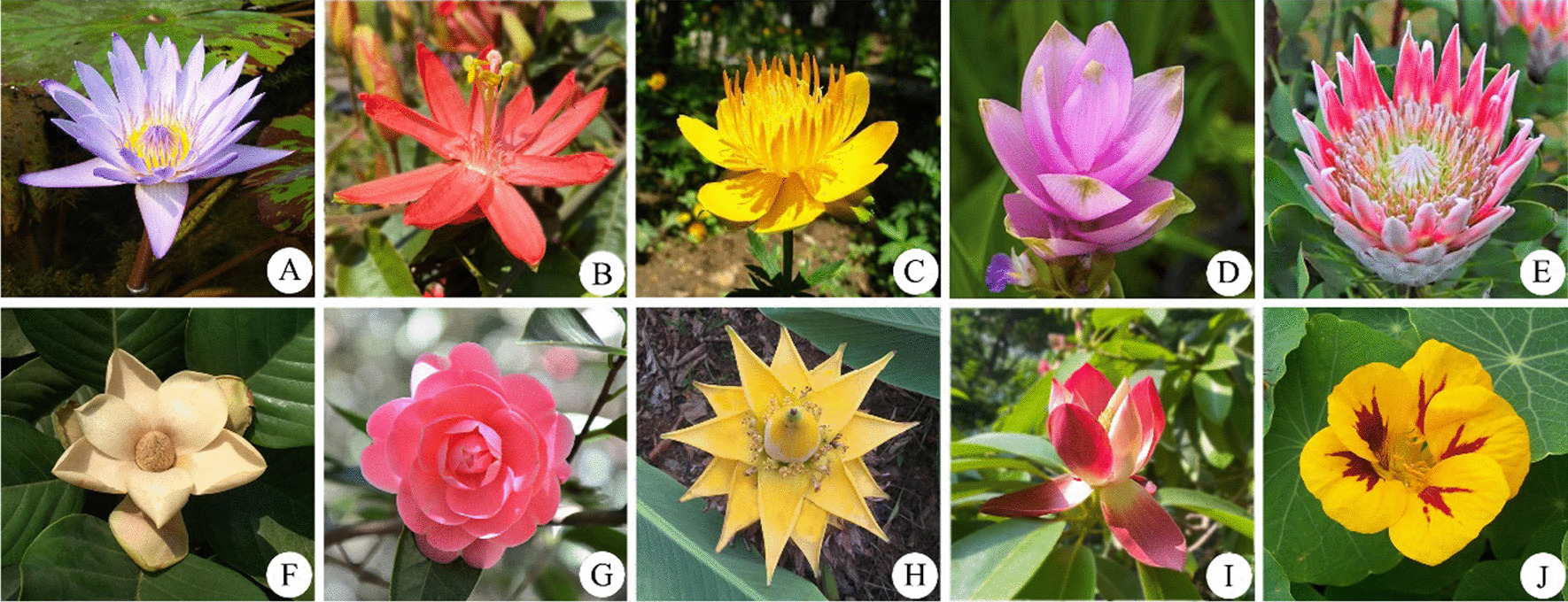
Ornamental plants associated with Buddha as lotus throne. **A** *Nymphaea tetragona*; **B** *Passiflora coccinea*;** C** *Trollius chinensis*; **D** *Curcuma alismatifolia*; **E** *Protea cynaroides*; **F** *Lirianthe delavayi*; **G** *Camellia reticulata* “Juban”; **H** *Musella lasiocarpa*; **I** *Manglietia insignis*; and **J** *Tropaeolum majus*

Buddha is often depicted sitting on a lotus throne while lecturing. The original lotus that Sakyamuni sat was the *Nymphaea tetragona* (Fig. 4A), which has rosette flowers and heart-shaped leaves, representing the symbol of love. Survey results showed 13 plants with lotus-like flowers or leaves were adopted to honor Buddha. For instance, *Nelumbo nucifera*, *Passiflora caerulea*, *P. coccinea* (Fig. 4B), and *Trollius chinensis* (Fig. 4C) replaced *N. tetragona* in N China. *Curcuma alismatifolia* (Fig. 4D) was substituted in S China and *Protea cynaroides* (Fig. 4E), *Lirianthe delavayi* (Fig. 4F), *Camellia reticulata* (Fig. 4G), *Musella lasiocarpa* (Fig. 4H), and *Manglietia insignis* (Fig. 4I) in SW China. The remaining *Tropaeolum majus* (Fig. 4J), *Clematis florida*, and succulent *Echeveria secunda* were adopted as a substitute for *N. tetragona* and to commemorate Buddha. Especially, the flowers of *L. delavayi* (Fig. 4F) and *M. lasiocarpa* (Fig. 4H) have a standing upright part in the middle, demonstrating Sakyamuni sitting on a lotus seat.

It has been suggested that Buddha’s bamboo monastery was the first Buddhist building, which later became a Buddhist temple. Although the original species used in the first bamboo monastery were unclear, some excellent bamboo plants were introduced around Buddhist temples named after bamboo plants. For example, Jin Zhu Temple in Chongqing was named after the bamboo *Phyllostachys sulphurea* (locally named “Jin zhu”, which means golden bamboo) with golden stalks. The Fang Zhu Temple in Lushan (Jiangxi Province) was named after the *Chimonobambusa quadrangularis*, with square stalks, called “Fang zhu” in Chinese, which means square bamboo. The Qiong Zhu Temple in Kunming was named after *C. tumidissinoda* with swollen nodes and is locally called “Qiong zhu”. Additionally, the *Senecio rowleyanus* was associated with Buddha because its leaves are comparable to Bodhi beads. Nevertheless, these ornamental plants are widely planted in parks, courtyards, and Buddhist temples, as well as indoors, to pray for blessings [3, 18–23].

### Ornamental plants associated with Bodhisattva

Thirteen ornamental plants were associated with Bodhisattva, including ten species with Guanyin, one with Maitreya with a swollen trunk, one with Manjushri, and one with Sangharama having a homophonic name.

**Fig. 5:**
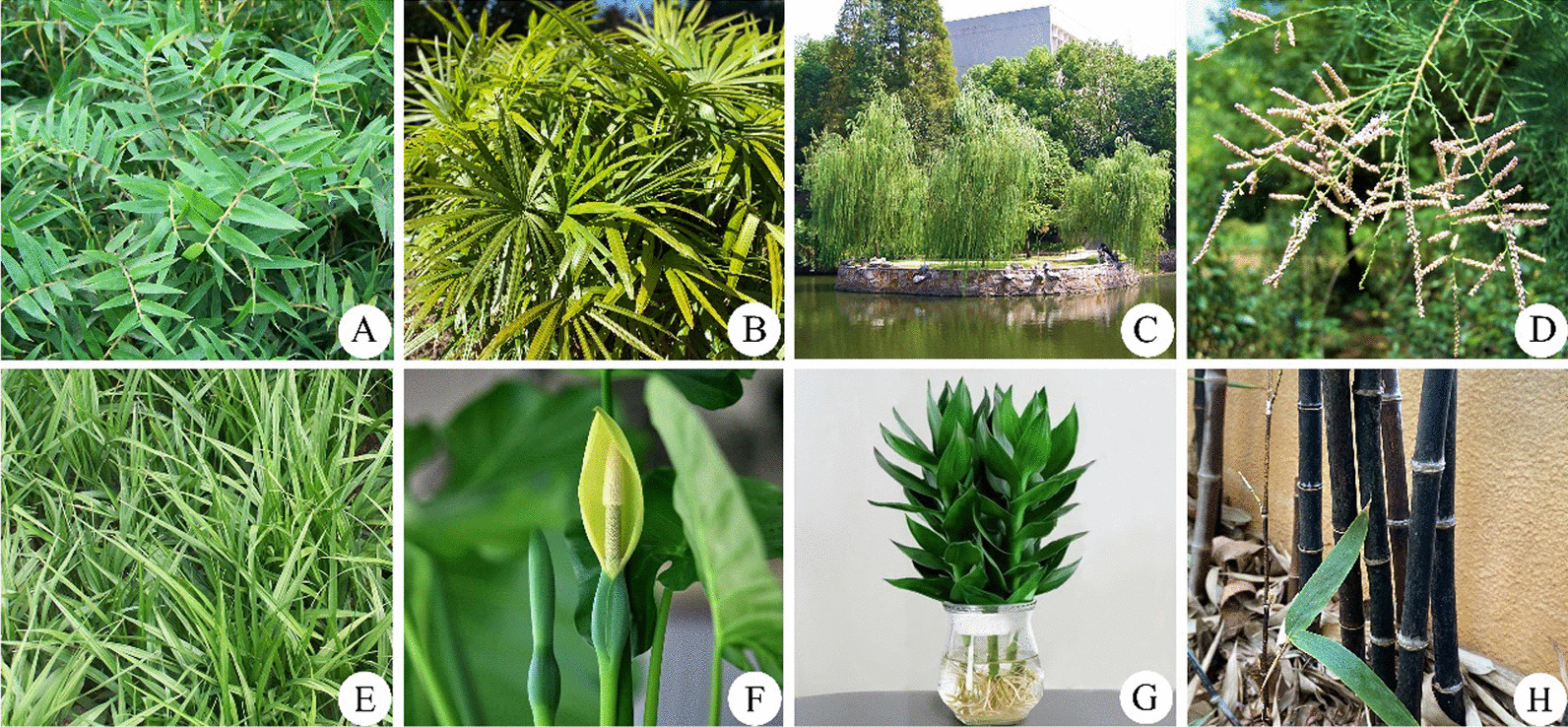
Ornamental plants associated with Bodhisattva Guanyin. **A** *Bambusa multiplex* var. *riviereorum*; **B** *Rhapis excelsa*; **C** *Salix babylonica*; **D** *Tamarix chinensis*; **E** *Reineckea carnea*; **F** *Alocasia macrorrhizos*; **G** *Crinum asiaticum* var. *sinicum*; and **H** *Gloriosa superba*

It is believed that the compassionate Bodhisattva Guanyin (known as the Bestower of Children in China) can save and liberate the victims by using the plant branches dip water in her holy bottle and then sprinkle drops of holy water to the human world [24]. The holy branches representing the compassion and softness in Guanyin’s bottle were generally the branches of *Salix babylonica* (Fig. 5C), *S.* *matsudana*, *Tamarix chinensis* (Fig. 5D), and the leaves of *Reineckea carnea* (Fig. 5E). The introduced species *Alocasia macrorrhizos* (Fig. 5F) was associated with Guanyin because its flowers look similar to Bodhisattva Guanyin. It was also believed that Guanyin preaches in the bamboo forest dominated by *Phyllostachys nigra* (Fig. 5H). The Zizhu temple in Kaohsiung (Taiwan Province, China) was named after *P. nigra* (Chinese name “Zi zhu”, which means purple bamboo). Additionally, the *Bambusa multiplex* var. *riviereorum* (Fig. 5A), *Rhapis excelsa* (Fig. 5B), and *Dracaena sanderiana* (Fig. 5G) were regarded as “Guan yin zhu”, which means Guanyin’s bamboo. The plant species associated with Guanyin have been planted around most Chinese Buddhist temples, as well as in parks, courtyards, and indoors [25, 26]. Particularly, the leaves from these plants are cut and used to decorate indoors [25, 26].

### Ornamental plants associated with Arhat

The Buddhist Arhat, pronounced “Luo han” in Chinese, means everything will succeed. The plants in the genus *Podocarpus* are called “Luo han song” in Chinese to honor Arhat because their seeds and seed stalks are morphologically similar to Arhat. Especially, *P. macrophyllus* and *P. forrestii* have been widely planted in Buddhist temples, urban green spaces, and courtyards and are also potted indoors to pray for wealth and honor [27]. For instance, two ancient *P. macrophyllus* trees were planted at the Lushan Temple in Changsha City (Hunan Province) when built about 1750 years ago.

### Evolving characters of the ornamental plants associated with Buddhist figures

Buddhist figures are popular and most admired in the daily life of Chinese people. Most of the ornamental plants associated with Buddhist figures were planted to admire both plants and Buddhist figures, and to pray for blessings, success, health, and wealth. Some important horticultural plants, such as *Annona squamosal* and *Sechium edule*, were associated with Buddhist figures mainly for promoting their commercial market.

Substitution of original plant species associated with Buddhist figures by native species with similar morphological characteristics is considered the main pattern of change for the original plants. For example, the heart-shaped leaves are considered the most prominent morphological feature of the original Bodhi tree, *Ficus religiosa*, and all nine local substitute species used to commemorate Buddha have heart-shaped leaves. Apart from the shape cues, the color yellow is also an important cue for the adaptation plants, of which 12 species have golden leaves, five species have yellow flowers, and three species bear yellow fruits. Additionally, plants with significant fragrance may help to be selected as the Buddhist figure associated species, such as *Curcuma alismatifolia*. Another adaptation is introducing non-native species and naming them based on comparable morphological characteristics to Buddhist figures. For example, *Jatropha podagrica* with swollen stem was introduced from Central America and named “Buddha’s belly tree”. As for the evolving time, the information on the ancient heritage trees in Buddhist temples provides the approximate ages of the beginning of their succession [28].

### Multiple use of ornamental plants associated with Buddhist figures

Among the ornamental plants associated with Buddhist figures, 33 (55.00%) were medicinal. *Ginkgo biloba* is the most commonly used medicinal plant, and the extract from its leaves is used to treat coronary heart disease, angina pectoris, and hyperlipidemia [29]. The *Siraitia grosvenorii* with Arhat-like roots has significant therapeutic effects on diseases like bronchitis and hypertension [30]. Some plant species were adopted for economic values. For instance, *S. grosvenorii* has been considered an important economic plant in Guangxi Province for many years because the extract of the species, mogroside, is 300 times sweeter than sucrose and can be used to make beverages [30]. Fifteen out of 60 species (23.33%), including *Annona squamosal*, *Sechium edule*, *Passiflora caerulea*, *Nelumbo nucifera*, *Musella lasiocarpa*, *Musa basjoo*, and bamboo species, have been planted for fruits and vegetables [19, 31]. Some species (*Syringa* spp. and *Tilia* spp.) have been planted and used as sources of perfume and nectar [11, 13]. Additionally, bamboo has been used to make papers, walking sticks, and artwork materials [32]. Utility aspects may influence the choices for certain plant species to be placed in Buddhist temples. For example, the species *Camellia reticulata* were cultivated in the many temples in central Yunnan 400 years ago due to its oil from seeds can be used as Buddhist lamp oil [22].

## Conclusions

In China, people grow ornamental plants associated with Buddhist figures to reflect their affection for both plants and Buddha, which aid in the inheritance of Buddhist culture and promote ornamental plants for commercial purposes. The current and successive Buddhist figure associated ornamental plants will play an important role in home gardening and landscape greening. The present study can serve as a basis for future investigation of modern Buddhist culture. It can also provide a reference for the construction of specialized ethnic botanical garden and theme park.

## Data Availability

Not applicable.
